# Stretchable Microelectrode Arrays for Electro‐Mechanical Probing of Brain Spheroids

**DOI:** 10.1002/adhm.71788

**Published:** 2026-10-06

**Authors:** Eleonora Martinelli, Ivan Furfaro, Flavius Timis, João Marques, Tala El Kaissi, Desirée Maulà, Laurine Kolly, Julian Bär, Sina Röllin, Scott Erickson, Laetitia Nikles, Benoît Delaup, Camille Delgrange, Loris Gomez Baisac, Luc Stoppini, Adrien Roux, Giuseppe Schiavone, Stéphanie P. Lacour

**Affiliations:** ^1^ Laboratory For Soft Bioelectronic Interfaces Neuro‐X Institute, École Polytechnique Fédérale de Lausanne (EPFL) Geneva Switzerland; ^2^ Tissue Engineering Laboratory HEPIA HES‐SO University of Applied Sciences and Arts Western Switzerland Geneva Switzerland

**Keywords:** brain spheroids, electrophysiology, kirigami electronics, mechanical actuation, multimodal systems, stretchable microelectrode arrays

## Abstract

Mechanical forces shape brain development and contribute to pathological conditions such as traumatic brain injury. However, their direct effects on neural electrophysiology remain difficult to elucidate. Here, we introduce a multimodal system capable of delivering controlled mechanical actuation to brain spheroids while simultaneously recording their spontaneous electrophysiological activity. The platform enables culturing spheroids onto kirigami‐patterned stretchable thin‐film microelectrode arrays over multiple days. The array reliably records spontaneous spheroid activity and sustains uniaxial mechanical stimulation with controlled elongation and speed, ranging from manual precision stretching to high‐velocity impacts. We capture spheroid deformation during slow stretch (∼100 µm/s) and detect acute electrophysiological changes following high‐velocity stimulation (∼1–2 m/s). This multimodal platform establishes a foundational system for systematic in vitro investigations of how mechanical forces regulate functional activity in 3D tissues.

## Graphical abstract

A multimodal device featuring kirigami‐patterned stretchable microelectrode arrays enables concurrent mechanical actuation and electrophysiological recording of brain spheroids. The platform supports multiple loading regimes and reveals mechanically induced modulation of spontaneous network activity in 3D neural tissue models.

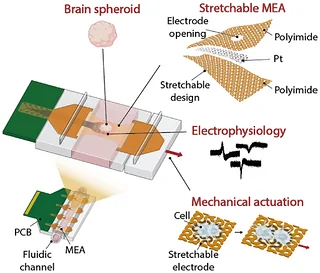

## 1 Introduction

Mechanical cues are fundamental regulators of cell behavior and are indispensable to the development and function of living systems. Across growth, differentiation, and morphogenesis, cells both generate forces and decode mechanical signals from their microenvironment [1]. In the central nervous system, these principles are expressed, among others, during brain development, where tissue folding and coordinated cell‐sheet movements impose localized physical stresses on neural progenitor cells that influence their fate and organization [2]. Mechanical perturbations also arise exogenously, most prominently in traumatic brain injury (TBI), in which an acute mechanical insult initiates dysfunction across cellular and circuit levels [3].

Understanding these relationships has motivated the development of in vitro platforms capable of delivering defined mechanical inputs to tissues with precise experimental control. Stretch‐based systems, where cells are cultured on deformable membranes subjected to prescribed strain, have been particularly valuable, as substrate deformation can be quantitatively related to the strain experienced by the tissue [4]. Stretch paradigms have been widely used to assess how deformation influences neural cell behavior [5, 6, 7], and analogous approaches have been adopted to recapitulate key aspects of TBI [8, 9].

Because neural function is ultimately mediated by electrical activity, models of injury and development increasingly require concurrent electrophysiological readouts. TBI is linked to alterations in electrophysiological dynamics [10], prompting the integration of mechanical loading platforms with microelectrode arrays (MEAs) to track neural activity before and after deformation (Table S1). Early implementations integrated a neuronal cell shearing injury device with a planar MEA [11], while subsequent work introduced stretchable MEA architectures that enabled mechanical interrogation of organotypic brain slices [12, 13, 14, 15]. More recently, Rogers and Gross proposed a concussion model using mouse embryonic cortical neurons cultured on a planar MEA subject to tangential acceleration, revealing rapid suppression of network spiking followed by partial recovery over acute timescales [16].

A persistent challenge in building informative in vitro models is the choice of tissue surrogate that accurately mimics the central nervous system. In recent years, brain organoids and spheroids have emerged as powerful human‐derived 3D models that provide a more physiological setting compared to 2D monolayers by allowing cells to self‐organize, form their own extracellular matrix, and establish 3D interactions and projections [17]. Importantly, growing evidence indicates that organoids are mechanosensitive, with exogenous mechanical modulation influencing patterning and morphogenesis through cytoskeletal remodeling and mechanotransduction pathways [18]. Moreover, organoid responses depend on both the magnitude and temporal dynamics of applied mechanical stimuli, as well as their interplay with the surrounding extracellular matrix [18]. In parallel, organoid‐based models are increasingly adopted in TBI research, particularly in compressive injury paradigms [19, 20, 21].

Human brain spheroids and organoids also exhibit spontaneous neural activity, creating an opportunity to directly connect mechanical perturbations to functional outcomes [22, 23]. Stretchable MEAs have been developed to conform to soft, curved 3D tissues and accommodate organoid geometry, enabling stable recordings from complex surfaces [24, 25, 26, 27, 28]. However, existing designs have prioritized conformability over active mechanical actuation, leaving a key technological gap: an experimental platform that can apply controlled mechanical stimulation to 3D neural tissues across distinct actuation regimes while maintaining a stable tissue–electrode interface for electrophysiological measurements.

Here, we address this need by engineering a multimodal system that integrates mechanical actuation of brain spheroids with concurrent electrophysiology and microscopy monitoring. Building on stretchable MEA concepts originally developed for 2D cultures [13], we adapt the approach to 3D neural models by enabling spheroids to adhere directly onto the perforated MEAs, ensuring recordings before, during, and after mechanical stimulation. Stretchability is engineered by design in the MEAs through micrometer‐scale Y‐shaped kirigami cuts in the polyimide/platinum stack [29, 30], yielding a perforated architecture with overall stretchability up to 10% uniaxial strain that also supports weeks‐long spheroid maintenance. The modular system accommodates uniaxial mechanical stimulation modalities ranging from slow elongation (∼100 µm/s) to high‐velocity impacts (∼1–2 m/s), while remaining compatible with microscopy and electrophysiology monitoring. As a proof‐of‐concept, we demonstrate spheroid deformation during slow stretching and an acute reduction in firing rate following high‐rate impacts. Taken together, our electro‐mechanical platform represents a first step towards the systematic investigation of how mechanical forces shape electrophysiological dynamics in 3D neural tissues. The platform has broad potential applications, ranging from studies of neurodevelopmental mechanobiology and in vitro models of TBI to other excitable tissue systems, such as cardiac organoids, where mechanics and electrophysiology are tightly coupled.

## 2 Results

### 2.1 Stretchable Kirigami‐Based MEAs

The core of the system is a stretchable MEA engineered to enable simultaneous electrophysiological recording and mechanical interrogation of 3D neural tissues. Unlike stretchable MEAs for 2D monolayers [31], devices intended for spheroids must accommodate 3D tissue maintenance. In particular, the limited diffusion of nutrients and oxygen to deeper tissue layers often results in necrotic core formation [32]. One established strategy to mitigate this constraint is air‐liquid interface (ALI) culture [33], where the tissue is maintained on a porous membrane, with one side exposed to air for oxygenation and the opposite in contact with culture media [34]. To enable ALI culture, stretchable MEAs need to be perforated. However, perforation geometry influences how strain is transmitted to the tissue. Because cells adhere to the substrate, substrate deformation results in deformation of the attached tissue. Large perforations can disrupt this mechanical coupling: when opening sizes approach the characteristic dimensions of the tissue [24, 25, 26, 27, 28], the tissue can deflect into the openings, resulting in reduced strain transfer in these regions. Micrometer‐scale perforations mitigate this effect and promote more uniform actuation of the tissue.

Taking into account these considerations, we implemented a kirigami‐inspired design [29, 30] in a polyimide/titanium‐platinum‐titanium/polyimide film stack. Both the platinum conductor (Pt, 200 nm‐thick) and the polyimide encapsulation (PI, 2 µm total thickness) were patterned with micrometer‐scale Y‐shaped cuts, generating a fully perforated MEA (∼80% porosity) including the electrode sites (Figure 1a). In our design, the kirigami hole‐patterns serve the dual purpose of supporting ALI culture and enabling mechanical stretchability. Importantly, both encapsulated conductors and unencapsulated electrode sites are kirigami‐patterned to allow mechanical actuation at all points, including directly at the electrode–tissue interface.

**FIGURE 1:**
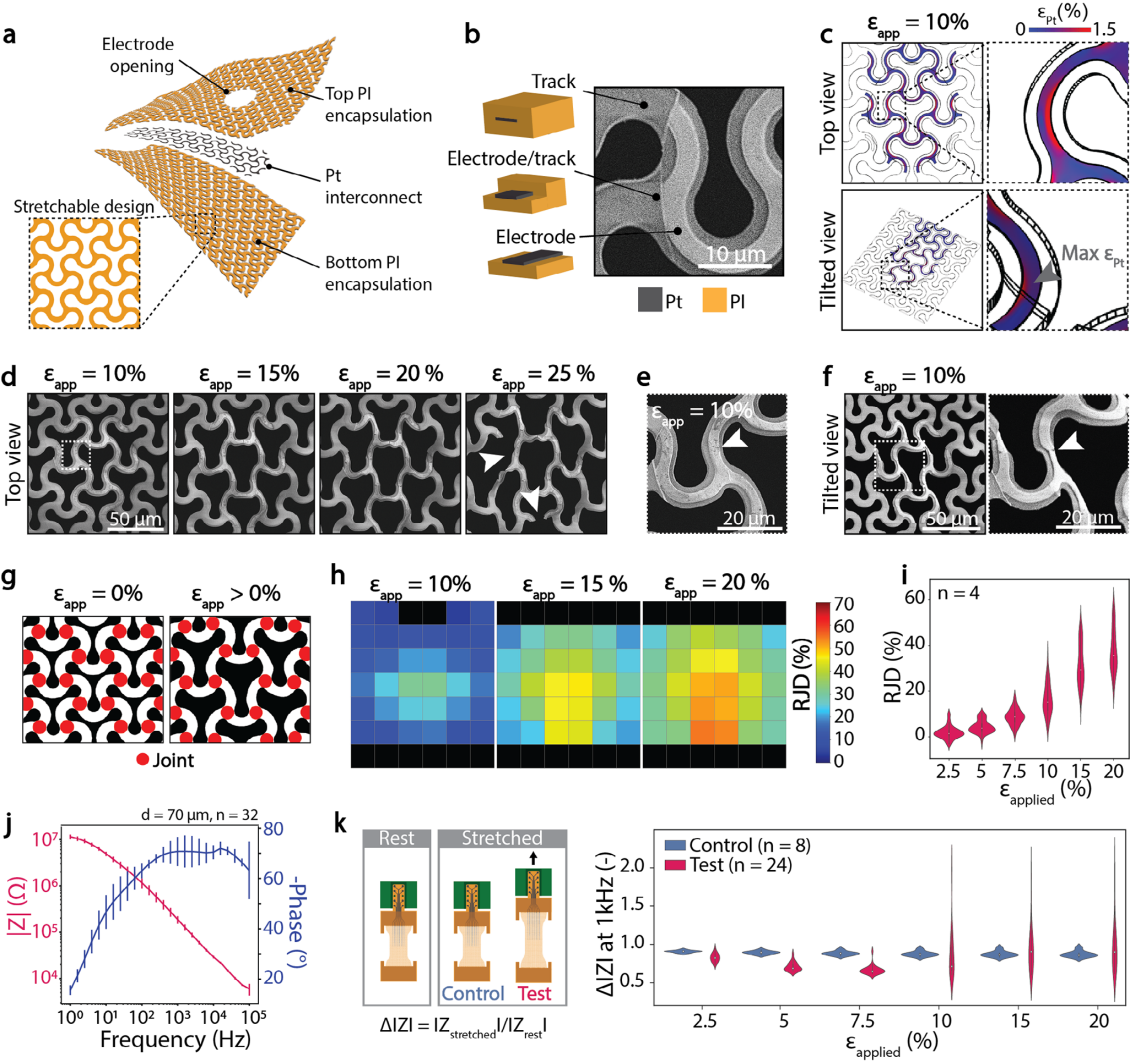
Stretchable kirigami‐based electrodes. (a) Exploded view of the stretchable electrode design consisting of a bottom PI encapsulation layer (1 µm), a Pt metallization layer (Ti/Pt/Ti 25/200/25 nm), and a top PI encapsulation layer (1 µm). The inset shows the Y‐shaped layout of the design. (b) SEM image of the electrode edge (right), and corresponding cross‐sectional schematics (left), highlighting the absence of top PI at the electrode opening and resulting in a sharp electrode/track transition. (c) FEM simulation of the strain distribution in the Pt layer of the electrode (ɛ_Pt_) under 10% overall strain (ɛ_app_). (d) In situ SEM images of the electrode undergoing increasing strain from 10% to 25% ɛ_app_. Arrowheads highlight mechanical failure at the electrode edge. (e) Close‐up SEM view of the electrode at 10% ɛ_app_ showing crack initiation in the Pt layer (arrowhead). (f) Tilted SEM view of the electrode at 10% ɛ_app_, highlighting Pt cracking at regions of curvature change associated with out‐of‐plane deformation (arrowhead). (g) Schematic representation of the joint positions used for RJD calculation. Joints correspond to the contact point of three strands in the Y‐shaped mesh and are tracked across strain levels. (h) RJD evolution for a representative electrode extracted from SEM image analysis (10% to 25% ɛ_app_). (i) RJD values of four electrodes under strain. (j) Electrochemical characterization of 70 µm‐diameter Y‐shaped Pt electrodes (*n* = 32). Error bars indicate standard deviation (SD). (k) Variation in |ΔZ| at 1 kHz as a function of ɛ_app_ for 24 stretched electrodes (three MEAs of 8 electrodes each, pink), compared to 8 unstretched controls (one MEA of 8 electrodes, blue).

In contrast to previous designs [29], in which kirigami‐patterned PI/Pt thin‐film interconnects were embedded within a polydimethylsiloxane elastomeric matrix to improve structural integrity and facilitate surgical handling, the mesh presented here is freestanding. While eliminating the supporting elastomer maximizes porosity, it also introduces handling challenges due to the thinness of the resulting structure. To enable reliable manipulation, the mesh was supported by a surrounding frame during handling and processing (Figure S1).

We first quantified the impact of the micrometer‐scale Y‐shaped kirigami patterning on the stretchability of a PI mesh with thickness matching the MEA encapsulation (2 µm). Consistent with previous reports [30], the kirigami cuts enabled reversible deformation in the PI mesh, with mechanical failure (defined as mesh rupture) occurring beyond 50% applied strain (ɛ_app_) and rupture loads below 0.5 N (Figure S2a–f). To enable the visualization of deformations at the microscale, we performed in situ tensile stretching under scanning electron microscopy (SEM) in a custom‐built setup (Figure S3). Out‐of‐plane deformation initiated at approximately 20% ɛ_app_, while residual strain was observed above 10% ɛ_app_, indicating the emergence of non‐linear deformation mechanisms (Figure S2g). These observations reveal how the microscale kirigami architecture accommodates large tensile strains through three‐dimensional deformation modes.

We next integrated electrode sites in the kirigami PI mesh. We selected a 70 µm kirigami electrode diameter to yield an effective electrochemical behavior comparable to that of conventional non‐stretchable ∼30 µm electrodes, commonly used for in vitro electrophysiology. Because electrode sites lack the top encapsulation layer, they introduce a sharp mechanical discontinuity between the surrounding kirigami PI and the unencapsulated electrode site (Figure 1b). To investigate the mechanical response of such a configuration, we performed finite element modelling (FEM) of single kirigami electrodes under tensile elongation and computed the resulting strain in the Pt layer. FEM predicted strain localization along the kirigami strands, with, unsurprisingly, peaks at the electrode edges (Figure 1c), consistent with the sharp electrode/track transition.

To experimentally validate these predictions, we fabricated dogbone samples comprising eight electrodes positioned along the y‐axis midline to minimize strain gradients (Figure S4) and performed in situ tensile stretching under SEM. Mechanical failure consistently initiated at the electrode edges (Figure 1d), in agreement with FEM strain maxima. Pt cracking appeared at ∼10% ɛ_app_ along the mesh strands, and co‐localized with curvature changes induced by out‐of‐plane deformation (Figure 1e,f). To quantify local deformation, we developed an image‐based analysis pipeline tracking mesh joint displacement and computing relative joint displacement (RJD), with RJD defined as the percentage change in distance between a joint and its neighbors compared to the initial state (Figures 1g and S5). In the 7.5–10% ɛ_app_ range, corresponding to crack onset, the electrode diverged markedly from the surrounding mesh, with electrode joints reaching up to ∼60% RJD prior to failure (Figure 1h,i).

Next, we electrochemically characterized the embedded kirigami electrodes. The electrochemical impedance of a 70 µm diameter kirigami Pt electrode (Figure 1j) was comparable to a 30 µm solid Pt electrode, in line with established literature [35]. To evaluate electrical functionality under mechanical deformation, we developed a dedicated testing setup (Figure S6) and measured the impedance magnitude |Z| at 1 kHz for stretched electrodes and compared it with unstretched controls (Figure 1k). We selected |Z| at 1 kHz because, as shown by the saline spectra (Figure 1j), the electrode response at this frequency is dominated by a capacitive phase. This makes |Z| at 1 kHz a measure of the electrode‐electrolyte interface capacitance, which should, in principle, stay unchanged under mechanical strain [36]. Starting at ∼10% ɛ_app_, however, |Z| increased and became more variable, indicating material degradation coinciding with Pt cracking.

Collectively, these results identify the electrode sites as the most mechanically vulnerable and failure‐prone regions of the kirigami‐based MEA, undergoing large local deformation under modest macroscopic strain (up to ∼60% RJD at ∼20% ɛ_app_). Crack initiation coincides with the onset of out‐of‐plane deformation and defines an electromechanical threshold beyond which impedance and recording stability begin to deteriorate. Kirigami electrodes show a branch‐by‐branch failure mechanism, which suggests that incorporating redundancy into electrode designs may delay total system failure.

Further analyses highlighted that both the electrode geometry (circular vs. rectangular) and the cross‐sectional geometry of the electrode/track transition (defined by the relative widths of PI and Pt, w_PI_/w_Pt_), influence stretchability (Figure S7). The proposed design, consisting of circular electrodes with w_PI_>w_Pt_, was selected to optimize mechanical performance. Electrode spacing and placement also affect strain distribution and the overall array stretchability (Figure S8). Deformation is anisotropic between the stretching axis and its perpendicular direction, and a minimum center‐to‐center spacing of ∼120 µm is required to prevent overlap of the deformation fields of 70 µm kirigami electrodes. Finally, electrode size further influences stretchability (Figure S9). During deformation, the affected region extends beyond the electrode footprint, with a deformed diameter ∼1.5x larger than the electrode diameter.

### 2.2 The Culturing Platform

To enable electrophysiological monitoring and mechanical probing of brain spheroids, we integrated the kirigami‐based MEAs into a compact culturing system designed to (i) minimize the system footprint to allow maintenance in a standard incubator, and (ii) enable transfer between culture, imaging, electrophysiology, and mechanical actuation without disturbing the tissue. These requirements motivated a modular device format (Figure 2a).

**FIGURE 2:**
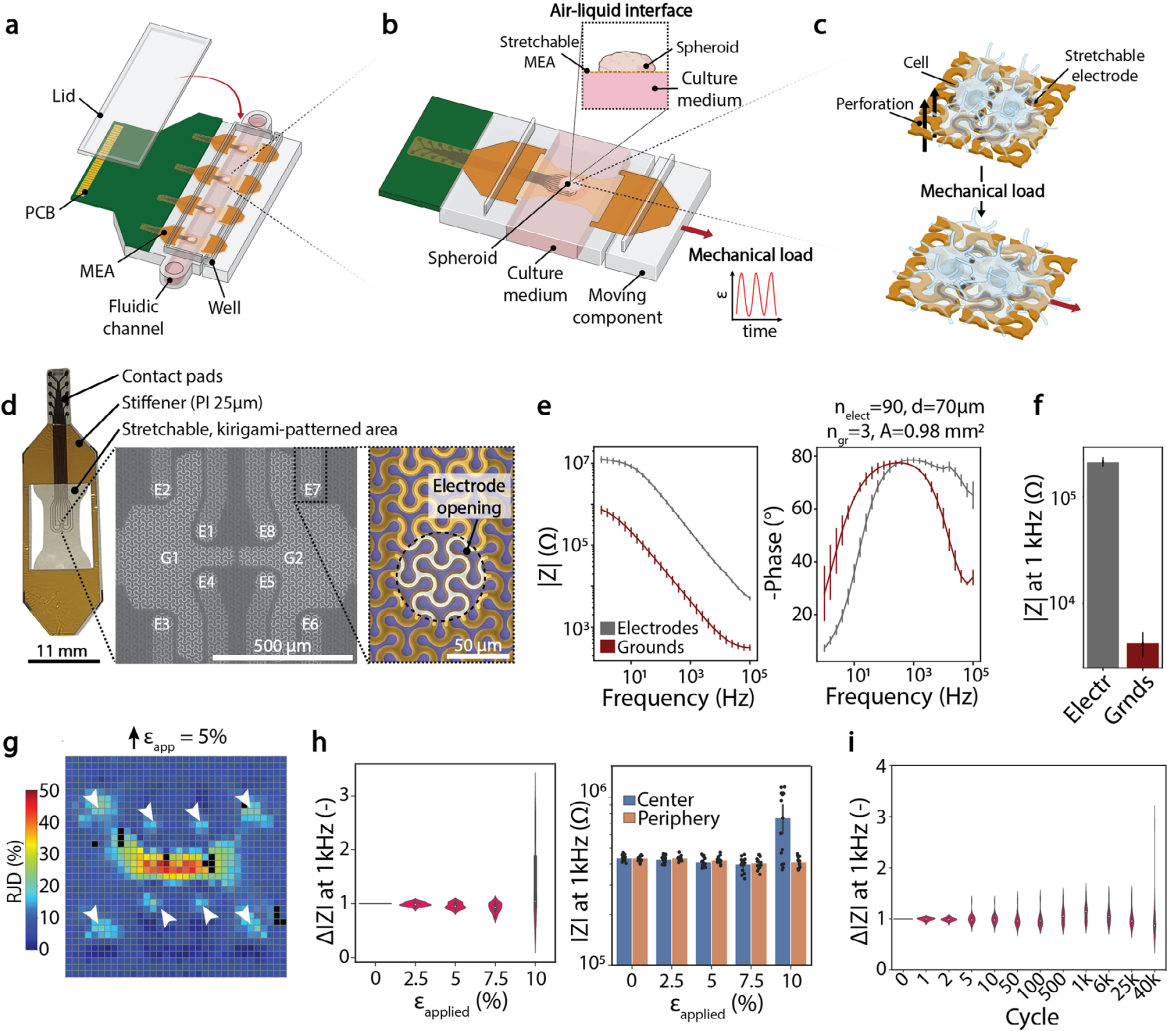
Integration of kirigami‐based MEAs into a spheroid culturing system. (a) Schematics of the device comprising four MEAs integrated with a fluidic channel for media exchange and connected to a PCB for electrophysiological recordings. (b) Schematic representation of a single MEA: the MEA hosts one spheroid, maintaining it at the air‐liquid interface; it is anchored to the PCB on one end and to a movable actuator on the other, enabling mechanical stretching. (c) Conceptual illustration showing that kirigami electrodes deform together with the tissue during loading, enabling electrophysiological readout under strain. (d) Left: photograph of a single MEA; middle: SEM of the kirigami‐patterned region of the MEA including eight recording electrodes (E1–E8) and two ground electrodes (G1–G2); right: SEM of a kirigami‐patterned interconnect and electrode with false‐color overlay indicating PI (yellow), Pt (white), and background (violet). (e) Impedance and phase spectra of the kirigami recording electrodes and grounds, with the grounds of each PCB shorted together (total: 3 devices, 90 electrodes, and 3 ground electrodes with 1 ground per device). Error bars indicate SD. (f) Extracted impedance magnitude at 1 kHz for recording and ground electrodes. Error bars indicate SD. (g) Relative joint displacement (RJD) map for a representative stretchable MEA at 5% ɛ_app_. Arrowheads indicate electrode positions. (h) Change in impedance magnitude at 1 kHz as a function of ɛ_app_ (left), and electrode position within the array (right). Center = [E1, E4, E5, E8], periphery = [E2, E3, E6, E7]. *n* = 4 MEAs. Error bars indicate 95% confidence intervals (CI) of the mean. **i** Change in impedance magnitude at 1 kHz at 5% ɛ_app_ as a function of the stretching cycle number over one week of automated cycling at 100 µm/s (*n* = 4 MEAs).

Building on established architectures [22, 37, 38] and to enable parallel measurements, we incorporated four MEAs into a single culturing device, with each MEA hosting a single spheroid and interfacing with a printed circuit board (PCB) for connection to the electrophysiology setup. To maintain the spheroids at the ALI, we suspended the MEAs over a fluidic channel assembled from transparent polymethyl methacrylate (PMMA; total thickness of 2 mm), allowing visualization by inverted microscopy (Figure 2b). We designed the overall configuration so that MEA actuation would directly translate into controlled spheroid deformation (Figure 2c), while remaining compatible with standard incubators and simplifying daily handling.

In each MEA, we patterned eight 70 µm‐diameter recording electrodes in a 0.5 mm^2^ grid to match the targeted spheroid dimensions (diameter 0.5–1 mm [39]), and we included two larger ground electrodes connected to the PCB ground plane [22, 37, 38] to serve as the electrical reference (Figure 2d). As expected, the ground electrodes showed lower |Z| at 1 kHz than recording electrodes, due to their larger size (Figure 2e,f).

As the MEA geometry critically influences strain distribution, we next verified the mechanical performance of the proposed configuration under tensile loading by elongating a kirigami‐based MEA under the SEM and mapping its corresponding RJD (Figure S10). At 5% applied strain, RJD mapping revealed a non‐uniform deformation field over the MEA surface with strain concentrating in the central region of the array between the two large ground electrodes, where it reached values as high as 50% (Figure 2g). From a functional perspective, the impedance magnitude of the recording electrodes remained stable during stretching up to 7.5% ɛ_app_ (Figure 2h), whereas at 10% ɛ_app_ the central electrodes exhibited the earliest impedance increases, consistent with localized damage. Finally, to assess durability under repeated actuation relevant for long‐term developmental studies (e.g., repeated stretching over multiple days), we subjected the kirigami‐based MEAs to 40 000 stretching cycles at 100 µm/s, corresponding to approximately one week of continuous actuation, and monitored their impedance evolution over time. Based on our prior electromechanical characterization, we selected 5% ɛ_app_ as a conservative regime for repeated actuation. As reported in Figure 2i, impedance remained stable across cycles for the majority of electrodes, with only one electrode exhibiting increased |Z| after 40 000 cycles, indicating robust performance and long‐term operability.

### 2.3 Brain Spheroid Integration and Electrophysiology Under Static Culture Conditions

Following the engineering of the culturing platform, we derived brain spheroids as previously described [39], and transferred them to the kirigami‐based MEAs. Given the spheroids' small size (<1 mm diameter), to ensure their accurate positioning over the MEAs, we adopted a membrane‐assisted transfer method reported by Govindan and colleagues using porous polytetrafluoroethylene (PTFE) membranes [39] (Figure S11). Following seeding, we maintained the spheroids over the kirigami‐based MEAs for multiple days (Figure 3a). SEM imaging after 8 days of culture on the device revealed qualitative evidence of integration between the brain spheroid tissue and the kirigami‐based MEAs (Figure 3b). Cellular processes were observed intertwining with the kirigami patterns, indicating close physical interaction. Over time, spheroids showed noticeable cellular outgrowth across the MEA surface (Figure 3c), accompanied by tissue flattening (Figure 3e). Histological observations confirmed the presence of both neurons and glial cells, with neurons predominantly localized in the spheroid core and glial cells dominating the outgrowth region (Figures 3d and S12).

**FIGURE 3:**
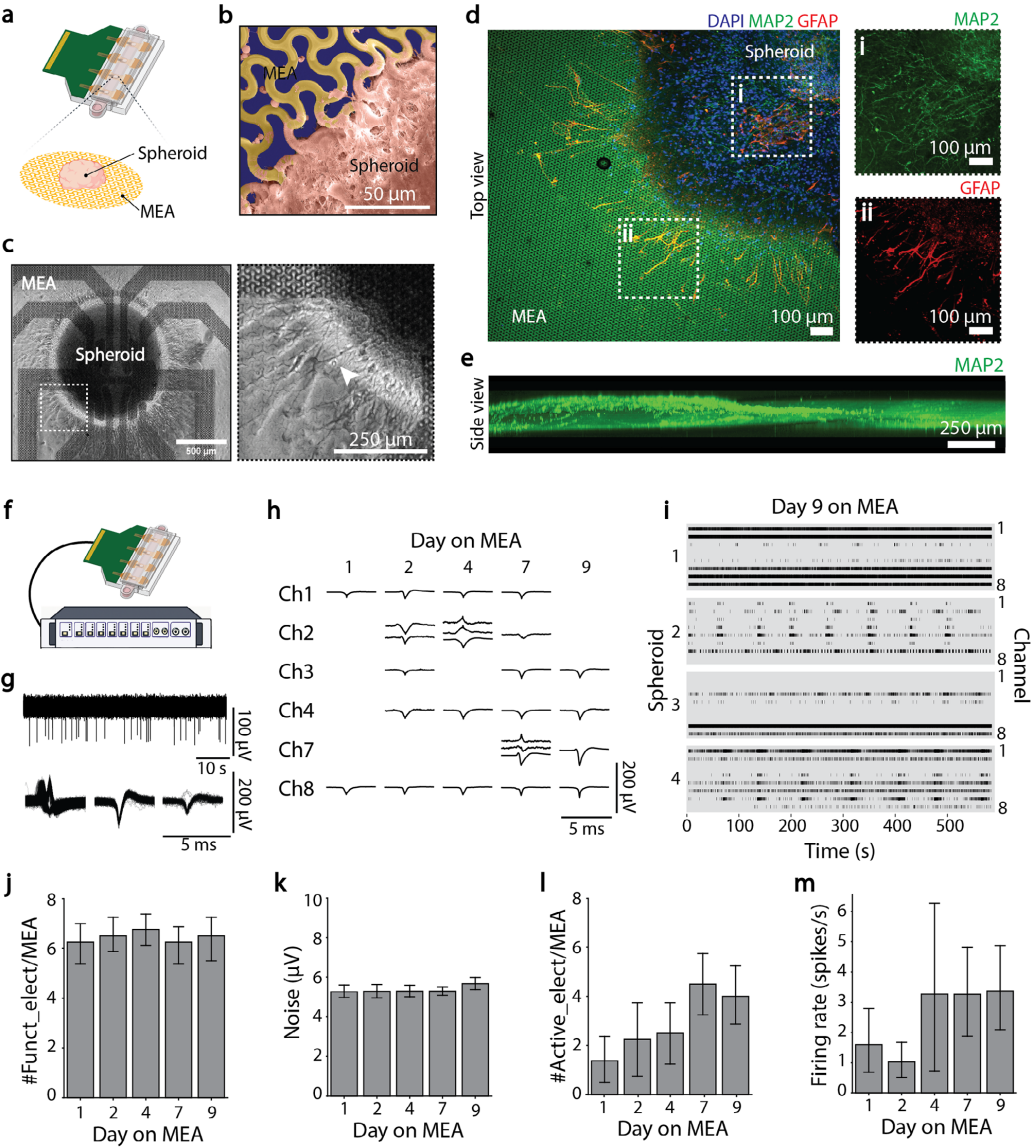
Spheroid integration with kirigami‐based MEAs. (a) Schematic representation of a biochip hosting a spheroid on an MEA. (b) SEM of a spheroid on the kirigami‐based MEA at 8 days post‐seeding, with false color overlay indicating the spheroid (pink), the MEA surface (yellow), and the background (blue). (c) Phase contrast images of a spheroid at day 9 on a stretchable MEA, illustrating cellular outgrowth extending around the spheroid contour (arrowhead). (d) Immunostaining of a spheroid on the stretchable MEA at 14 days post‐seeding, showing MAP2^+^ neurons (green), GFAP^+^ glial cells (red), and DAPI‐stained cell nuclei (blue). (e) Z‐projection of the MAP2 signal of a spheroid on the stretchable MEA at 14 days post‐seeding. (f) Schematics of the biochip connected to the electrophysiological recording system. (g) Representative electrophysiological recording of spontaneous neural activity from a kirigami electrode at day 1 post‐seeding (top; 1 min trace), and overlay of the detected neural events from three representative channels detected during 10 min‐long recordings (bottom). (h) Mean spike waveforms detected by six representative channels of a kirigami‐based MEA in 10 min‐long recordings as a function of day post‐seeding. (i) Raster plot of spontaneous neural activity from a representative device hosting four spheroids at day 9 post‐seeding (*n* = 4 MEAs, 4 spheroids). (j) Number of functional electrodes (noise level < 10 µV) per MEA as a function of time (*n* = 10 MEAs, 10 spheroids). (k) Noise level of functional electrodes over time (*n* = 10 MEAs, 10 spheroids). (l) Number of active electrodes (noise level < 10 µV and spike rate > 2 spikes/min) per MEA over time (*n* = 10 MEAs, 10 spheroids). (m) Quantification of the brain spheroid firing rate over time across all active electrodes (*n* = 10 MEAs, 10 spheroids). Error bars indicate 95% CI of the mean.

To gain insights into the neural behavior of the spheroids on the kirigami‐based MEAs, we monitored their spontaneous electrophysiological activity over multiple days post‐seeding (from day 0 to day 9). Recordings were acquired in situ inside the incubator to preserve culture conditions, and neural events were identified using a threshold‐based detection method. Representative band‐pass filtered traces show clear spike‐like events (Figure 3g), demonstrating that the kirigami electrodes reliably capture spheroid electrophysiological activity. Consistent with previous reports in comparable neural tissues [38, 39], neural events exhibited both positive and negative peaks with amplitudes typically ranging between ∼30 µV and 100 µV (Figure 3g,h). Spontaneous activity included both continuous spiking and periodic bursting‐like patterns (Figure 3i), in agreement with electrophysiological signatures previously reported for similar 3D neural models [37, 38, 39]. Based on observation, we classified the recording channels as *functional* when their noise level was < 10 µV, and *active* when their detected spike rate exceeded 2 spikes/min. The number of functional electrodes per MEA remained stable throughout the observation period (Figure 3j), with an average noise level of ∼5.5 µV (Figure 3k), indicating robust electrical performance of the kirigami arrays. While the electrodes were largely functional, only ∼1.5 electrodes per MEA were active at day 1, with activity increasing only modestly over time (Figure 3l). At the population level, mean firing rates increased from ∼1–2 spikes s^−^^1^ on day 1 to ∼3–4 spikes s^−^^1^ by later time points (Figure 3m), potentially reflecting progressive tissue remodeling and functional reorganization at the MEA interface. Overall, spiking behavior was dynamic across electrodes and over time, with waveform properties and firing patterns evolving.

### 2.4 Case Study: Electrophysiology During Mechanical Actuation

Next, we demonstrated that kirigami‐based MEAs enable simultaneous deformation of brain spheroids and recording of electrophysiological activity, allowing electro‐mechanical interrogation of 3D neural tissues. To do so, we investigated two complementary actuation modalities: (i) slow stretching, relevant to developmental‐scale mechanical cues, where controlled deformation at defined strain levels was performed during imaging and electrophysiological recordings, and (ii) fast actuation, designed to approximate high‐velocity deformation associated with traumatic injury.

For slow actuation, we developed a uniaxial stretching system compatible with both electrophysiological recordings and optical microscopy (Figure 4a). We based the stretcher on a precision linear stage actuated via a manual handle, enabling controlled elongation at low strain rates (∼100 µm s^−^^1^), and readily adaptable to motorized actuation in future iterations. We mounted the culturing device on an alignment support to ensure reproducible positioning (Figure S13a), and we performed stretching either directly on this support or on an inverted microscope stage, enabling simultaneous imaging of tissue deformation (Figure S13b). To confirm strain transfer from the MEA to the tissue, we labeled spheroids with a membrane dye and performed live imaging during elongation. Representative images show progressive tissue deformation with increasing applied strain, with an ∼18% tissue elongation along the stretching axis at 10% ε_app_ and ∼7% contraction in the orthogonal direction (Figures 4b,c and S14). Although not directly quantifying adhesion strength of the spheroid to the kirigami MEA, these observations indicate that mechanical coupling was sufficient to transmit at least part of the applied strain to the spheroids.

**FIGURE 4:**
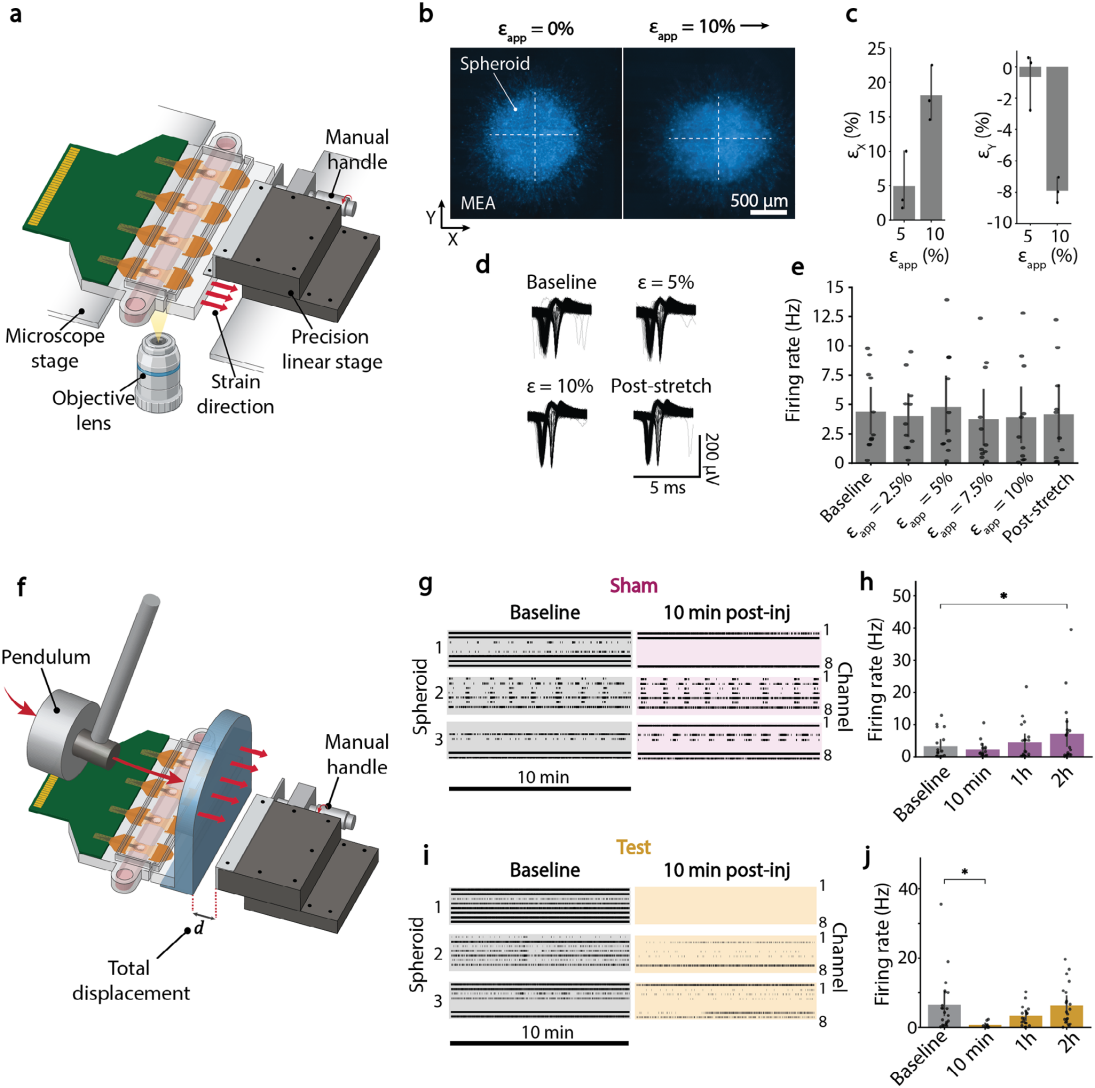
Electro‐mechanical probing of brain spheroids. (a) Schematic of the low‐rate stretching setup: the movable element of the fluidic channel is actuated using a manually operated linear stage (handle‐driven). The system is compatible with inverted microscopy, enabling simultaneous imaging of spheroid deformation during elongation. (b) Cellular membrane‐stained spheroid on a kirigami‐based MEA at ɛ_app_ = 0% and ɛ_app_ = 10%. (c) Measured spheroid elongation along *x* and *y* axes (ɛ_X_, ɛ_Y_). Error bars indicate 95% CI of the mean. (d) Overlay of spike events detected from a representative channel during 10 min recordings acquired at increasing strain levels. (e) Firing rate as a function of the applied strain, from ɛ_app_ = 0% to ɛ_app_ = 10% (*n* = 2 MEAs, 2 spheroids). Dots: active electrodes per strain level (11 for baseline and ɛ_app_ = 2.5% – 10%, 10 post‐stretch). Error bars indicate 95% CI of the mean. (f) Schematic of the pendulum‐actuated high‐rate stretcher: the movable element of the fluidic channel is coupled to a linear stage driven by the striker arm of a pendulum; total displacement *d* is adjusted to set the targeted strain. (g) Raster plots of the spontaneous electrophysiological activity recorded during the sham condition at day 9 post‐seeding at baseline and 10 min after sham injury (*n* = 3 MEAs, 3 spheroids). (h) Firing rate in the sham condition at baseline and following injury. *p < 0.05, paired two‐tailed *t*‐test comparing each post‐injury time point with baseline (14 paired active electrodes at 10 min, 18 at 1 h, and 17 at 2 h). Dots: total number of active electrodes per time point (18 at baseline, 15 at 10 min, 20 at 1 h, 19 at 2 h). Error bars: 95% CI of the mean. (i) Raster plots of the spontaneous electrophysiological activity of three brain spheroids recorded in the test condition at baseline and 10 min after trauma. (j) Firing rate in the test condition at baseline and following injury. *p < 0.05, paired two‐tailed *t*‐test comparing each post‐injury time point with baseline (10 paired active electrodes at 10 min, 18 at 1 h, and 21 at 2 h). Dots: total number of active electrodes per time point (21 at baseline, 10 at 10 min, 18 at 1 h, 21 at 2 h). Error bars: 95% CI of the mean.

We next assessed whether kirigami‐based MEAs maintain electrophysiological recording performance during elongation. We performed experiments at day 9 post‐seeding, a time point empirically selected to balance a sufficient number of active electrodes with a manageable experimental duration. We elongated two MEAs within the same device stepwise from ε_app_ = 2.5% to 10%, acquiring 10‐min recordings at baseline (ε_app_ = 0%), at each strain level, and after relaxation back to ε_app_ = 0% (Figure S15a,b). We carried out all recordings inside an incubator to preserve tissue viability: for each strain condition, we briefly opened the incubator to manually adjust the strain (steps of ε_app_ 2.5%, corresponding to ∼ 300 µm displacement over ∼ 3s), then performed the 10‐min recording under stable incubation conditions with the MEA held at the target strain. The number of functional electrodes remained stable across all strain levels (Figure S15d), with noise levels maintained at ∼6 µV (Figure S15e), indicating robust MEA recording performance throughout mechanical deformation. Spiking activity was detected before, during, and after elongation (Figure 4d,e). While raster plots revealed fluctuations in activity at the channel level during stretching (Figure S15c), the fraction of active electrodes (Figure S15f) and the overall firing rate (Figure 4e) did not significantly change, suggesting that slow elongation did not strongly alter global spheroid electrophysiology under these conditions.

To model injury‐relevant deformation, we further engineered a compact stretch–injury platform actuated by a pendulum striker arm (Figure 4f). Because concussive events are associated with impact velocities on the order of ∼1–2 m/s [40] that are difficult to reproduce with conventional cell‐culture stretchers, we used a pendulum‐based design to generate high actuation speeds in a benchtop‐compatible format. We tuned displacement, velocity, and force by adjusting key system parameters (Figure S16), providing reproducible control over high‐rate mechanical loading. Motivated by repetitive impacts typical of contact sports [40], we implemented a trauma protocol consisting of 10 consecutive impacts at ε_app_ = 7.5%, delivered every 30 s to three MEAs. This strain range remains relevant, as deformations as low as 5% have been reported to affect neuronal function in hippocampal slice cultures [41]. To account for variability in spontaneous electrophysiological dynamics, we conducted trauma experiments at day 9–10 post‐seeding, after spheroids had adapted to the culturing environment for >1 week. We implemented both *test* and *sham* conditions (Figure S17a). In the test condition, we recorded baseline activity before trauma and repeated recordings at 10 min, 1 h, and 2 h post‐actuation, consistent with time scales reported in injury recovery studies [22, 23]. In the sham condition, we removed and returned the device to the incubator without mechanical actuation to control for handling and temperature effects [42]. In both conditions, the number of functional electrodes remained stable (Figure S17c), confirming recording reliability. Notably, the test condition exhibited a significant reduction in firing rate 10 min after trauma (Figure 4i,j), consistent with an acute suppression of neural activity induced by high‐rate mechanical deformation [22]. In contrast, firing rate did not significantly change at 10 min in the sham condition (Figure 4g,h), indicating that the observed early electrophysiological response was primarily driven by mechanical trauma. Nevertheless, a transient reduction in the number of active electrodes was observed in one sham spheroid (Figure S17d), likely due to changes in temperature, CO_2_, and/or humidity while outside the incubator. Overall, these results point to measurable electrophysiological sensitivity to fast mechanical actuation, while larger cohorts will be required to rigorously quantify variability and establish statistical robustness.

## 3 Conclusion

In this work, we developed a stretch‐based multimodal system enabling simultaneous mechanical stimulation and electrophysiological recording of brain spheroids, addressing a key limitation in current toolkits for 3D neural models. Although kirigami‐based MEAs have previously been reported for three‐dimensional electrical interfacing [25], and 2D mechanical stretch systems are well established [43, 44], these approaches have not been integrated into a unified platform for controlled electromechanical interrogation of 3D spheroids. Here, we bridge this gap by combining a kirigami MEA with mechanical actuation, enabling simultaneous recording and deformation of 3D neural tissues.

The kirigami architecture of the engineered MEAs supported ALI culture and weeks‐long tissue maintenance, and provided programmable stretchability through micrometer‐scale Y‐shaped cuts. Within this framework, the MEA not only allowed for the recording of neural activity but also served as an active tool for controlled tissue deformation. Electromechanical characterization of the kirigami‐based MEAs revealed strain localization at electrode openings, with Pt cracking at ∼10% ɛ_app_ coinciding with impedance change, identifying the electrode–track transition as the primary failure site and a key design constraint. The kirigami‐based MEAs reliably recorded spontaneous spheroid activity over multiple days, capturing spike‐like events (∼30–100 µV) with stable noise levels (∼5.5 µV), thereby validating patterned, perforated electrodes for electrophysiology. Thanks to its modular design, the platform supported uniaxial mechanical actuation across regimes spanning slow elongation (∼100 µm/s) to high‐velocity impacts (∼1–2 m/s). As a proof‐of‐concept, we observed spheroid deformation during stretching and an acute reduction in firing rate after high‐rate loading, demonstrating the platform's potential to study mechano‐electrophysiological coupling and injury‐like dynamics in 3D neural tissues.

Compared with intrinsically stretchable MEAs reported for neural organoids, which achieve larger strain ranges [26, 27], the present design prioritizes higher patterning resolution (∼6 µm width of each MEA strand and ∼2 µm width of Pt branches) at the expense of maximal stretchability. This trade‐off underscores a central consideration for future platforms: the need to balance culture compatibility, mechanical compliance, and electrical performance across intended deformation regimes. In the current implementation, the electrode site represents the mechanical weak point, introducing new reliability challenges as the device transitions from electrophysiological recording to driving mechanical actuation. Continued optimization of kirigami geometries, conductor layouts, and material stacks may extend functional strain ranges while preserving signal fidelity.

A limitation of the current implementation is that electrophysiological recordings are primarily obtained from the organoid surface. Future designs could enable more spatially distributed interrogation of the tissue volume by tuning the kirigami mesh geometry to allow tissue penetration and encapsulation of the electrode array, as demonstrated in previous bioelectronic interfaces [45]. Such approaches could facilitate recording from deeper regions of the organoid while preserving controlled mechanical deformation.

From a mechanical actuation perspective, the choice of stretching mechanism determines the achievable strain rates and, consequently, the physiological relevance of the applied loading. Whereas conventional stretchers are generally limited to low‐to‐moderate strain rates [43, 44], higher‐rate systems often require bulky and specialized equipment [9]. To address this gap, we developed a modular platform incorporating a pendulum‐based actuator capable of delivering rapid mechanical loading in a compact tabletop format. Although the resulting loading environment may involve additional effects, such as shear forces and vibrations, which warrant further characterization, this approach expands the range of accessible loading regimes without complex infrastructure. For slow, controlled elongation, the proposed platform could be further equipped with a motorized actuation system in future iterations, enabling simultaneous MEA stretching at a defined strain rate and electrophysiological recording while the MEA is in motion. Such an implementation would, however, require careful consideration and mitigation of potential motion artifacts during recording.

At the system level, the successful integration of MEAs with fluidic components, PCBs, and actuation modules demonstrates the feasibility of assembling complex, multimodal functionalities into a unified platform. This represents a significant advancement toward more comprehensive in vitro models; however, it also introduces fabrication and assembly challenges associated with interfacing heterogeneous components. Addressing these challenges through simplified device architectures and streamlined assembly workflows will be critical for improving robustness, scalability, and experimental throughput.

From a biological standpoint, the present study focuses on spontaneous neural activity, which is inherently variable and may limit sensitivity in detecting functional changes following mechanical perturbation. Future studies will benefit from incorporating evoked activity paradigms, such as chemical [12] or electrical [14] stimulation, to improve the detection of subtle functional changes. Extending observation windows will be important to capture delayed and long‐term effects of mechanical loading on tissue health and network dynamics [22]. In addition, the use of graded injury paradigms (e.g., single vs. repeated impacts) will provide further insight into potential dose‐dependent effects. In parallel, combining electrophysiological measurements with spatial mapping of strain distributions within the 3D tissue will enable more precise interpretation of mechanobiological responses. Integrating complementary biological readouts, including secondary injury biomarkers (e.g., oxidative stress markers [46]) and pharmacological interventions [47], will further strengthen mechanistic insights into trauma‐induced network alterations.

Although validated here using brain spheroids, the platform is readily extendable to more complex 3D brain models, offering a path toward more physiologically relevant in vitro studies of brain mechanobiology and traumatic injury. Beyond neural models, the platform could be broadly applied to other electrically active 3D in vitro tissues, such as cardiac organoids, where controlled mechanical stimulation may promote tissue maturation and recapitulate disease‐relevant forces, while enabling simultaneous functional assessment [48, 49]. In this way, this approach lays the groundwork for a new generation of in vitro platforms that more closely bridge engineered tissues with the dynamic complexity of living systems.

## 4 Experimental Section/Methods

### 4.1 Finite Element Modelling of the Kirigami Electrode

Simulations were carried out using the Solid Mechanics module in COMSOL Multiphysics (version 6.0). The 3D geometry of single kirigami electrodes was constructed using the built‐in design tools available in the software. The kirigami electrodes had a 70 µm diameter and were positioned at the center of a 150·150 µm^2^ kirigami PI membrane. The electrodes were composed of three layers: a 1‐µm‐thick PI substrate, a 200 nm‐thick Pt metallization layer, and a 1‐µm‐thick PI encapsulation layer. To enable the model to converge to a buckled state, a geometric perturbation was introduced in the form of a sinusoidal in the *xz* plane *z = 0.05·sin(4π·x/30.022)*, where *x* and *z* were the x‐ and z‐coordinates, respectively. Materials were modeled as linear elastic: PI was modeled with density (*ρ*) 1300 kg/m^3^, Young's modulus (*E*) 3.1 GPa, and Poisson's ratio (*ν*) 0.34; Pt with *ρ* = 21 450 kg/m^3^, *E* = 168 GPa, and *ν* = 0.38. The built‐in “fine” free triangular mesh was used. One side wall of the membrane was fixed in all directions. On the lateral side walls of the membrane, the displacement was set to zero along the x and z axes. A displacement boundary condition was imposed on the remaining side walls to induce mechanical deformation of the membrane. A stationary solver was used for the static analysis of strain and stress under a prescribed displacement, applied using a stepwise parametric sweep. Nonlinear geometry effects were activated to account for large deformations. The initial displacement was set to zero. The first principal strain in the Pt layer upon elongation was extracted from the simulation results. The reported strain maps exceed the plastic strain limit of Pt and are presented solely for visualization to identify regions of strain concentration.

### 4.2 Kirigami‐Based MEA Fabrication

The fabrication process is schematized in Figure S1. The process started with the deposition of a 1 µm layer of polyimide (PI; PI‐2610, HD MicroSystems) via spin‐coating on a 4‐inch silicon (Si) wafer previously coated with a Ti/Al sacrificial layer (20/100 nm). Following treatment of the PI surface with O_2_ plasma, a Ti/Pt/Ti metallization layer was sputtered (Pfeiffer SPIDER 600), with a thickness of 25/200/25 nm. To define the metallic interconnects, the metal was coated with a 5.5 µm layer of photoresist (ECI3027, Merk), which was subsequently patterned via direct laser writing (MLA 150, Heidelberg Instruments), followed by metal dry etching (210IL, Corial). After treating the PI surface with O_2_ plasma at 200 W for 30 s (V10‐G, PINK), an additional 1 µm layer of PI was spin‐coated on the wafer to encapsulate the metallic tracks. Electrodes and pads were defined by spin‐coating a 5.5 µm photoresist layer (ECI3027, Merk), which was subsequently patterned with photolithography to expose the contact sites (MLA 150, Heidelberg Instruments). The 1 µm PI top encapsulation layer over the electrodes and pads was then etched, followed by the etching of the top 25 nm Ti layer of the contact metallization. A third photolithographic step was performed to define the device outline by coating and patterning a 5.5 µm photoresist layer (ECI3027, Merk), followed by PI etching. Different designs were used depending on the considered experimental condition. Stiffeners were created by laminating a 25 µm Kapton sheet onto double‐sided tape and patterning via laser micromachining (WS Turret, Optec Laser Systems). The stiffeners were then manually laminated over each MEA. The MEAs were released from the Si carrier wafer by anodic dissolution of the Al sacrificial layer in a saturated NaCl solution, as previously described [50]. To improve handleability, a polydimethylsiloxane (PDMS) support with a thickness of ∼1.5 mm was placed over the pads of the MEAs and used to move the MEAs and align them with the PCB contacts. Soldering paste (NP510‐LT HRL1 low temperature solder paste, Kester) was screen printed on the contacts of a rigid PCB, followed by alignment of the MEAs using a custom‐made aligner and soldering by heating (∼190°C for 3 min). Finally, the contact area between the MEA and the PCB was sealed in flowable sealant (734, Dow Corning) and cured at room temperature overnight.

### 4.3 Device Assembly

The MEAs and PCBs were subsequently integrated into a custom‐built polymethyl methacrylate (PMMA) platform. Briefly, PMMA sheets of various thicknesses (Southern Acrylics) were laminated with double‐sided tape (130 µm‐thick Double Coated Tapes with Laminating Adhesive 300LSE, 3 M), laser cut (Trotec Speedy 100), and assembled. The fluidic chamber had a total volume of 1 mL. Before tissue seeding, mounted biochips were sterilized in UV‐ozone (steriLux).

### 4.4 Scanning Electron Microscopy Under Uniaxial Elongation

Scanning electron micrographs of the kirigami electrodes were acquired (SU5000, Hitachi) while stretching the MEA with a custom‐built stretcher that controlled the distance and speed of the clamps. To allow for reproducible sample placement, the MEAs were fixed on the clamps of the stretcher using a custom‐built holder, as illustrated in Figure S3. Each MEA was uniaxially stretched, symmetrically, with a speed of 100 µm/s and a step size of 306 µm (ε_app_ = 2.5%).

### 4.5 Relative Joint Displacement (RJD) Computation

The analysis pipeline developed to generate RJD maps is schematized in Figure S5. Image processing and analysis were performed in Python (v3.11.9) using custom scripts. RJD quantification was performed on the SEM images acquired during uniaxial stretching. For each sample, one *reference* image was acquired at ε_app_ = 0% and a set of *target* images at increasing strain levels (ε_app_ > 0%). First, all SEM images were segmented and converted into binary masks to isolate the kirigami mesh (white) from the background (black). We then identified mesh *joints* as the intersection points between three kirigami strands. This yielded a set of joint coordinates (*x*, *y*) in the reference image and in each corresponding target image. To establish correspondence between joints across deformation states, we performed joint tracking by matching each joint in the target image to its homologous joint in the reference image (visual verification was performed using color‐coded overlays). To compute local deformation in a consistent manner, we generated a grid‐based neighborhood assignment in which each grid cell contained a single joint. This enabled identification of the nearest “up” and “down” neighbors for each joint along the Y direction (i.e., the stretching axis). For every joint, we computed the vertical distances to its up and down neighbors in both the reference and target images, yielding *d_ref, up,Y_*, *d_ref, dw,Y_*, *d_trg, up,Y_*, and *d_trg, dw,Y_*. We then calculated the normalized distance changes (in %) as:

$$\begin{array}{cc} & \;RJD_{up}=100\cdot \frac{\Delta \left(d_{trg,up,Y},\;d_{ref,up,Y}\right)}{d_{ref,up,Y}},\;\\ & \;RJD_{dw}=100\cdot \frac{\Delta \left(d_{trg,dw,Y},d_{ref,dw,Y}\right)}{d_{ref,dw,Y}}. \end{array}$$

Finally, we defined the relative joint displacement for each joint as the mean of the two values:

$$RJD=mean\left(RJD_{up},RJD_{dw}\right).$$

### 4.6 Electrochemical Characterization

All measurements were performed in 1X phosphate‐buffered saline (PBS) solution at room temperature, in a three‐electrode setup with an Ag/AgCl reference electrode and a large‐area stainless steel rod as the counter electrode. The MEAs were immersed in PBS solution. The electrochemical impedance was recorded with an impedance analyzer (Autolab PGSTAT302N, Metrohm) for frequencies between 1 Hz and 100 kHz, measuring five points per decade. Electrodes with impedance amplitude above 500 kΩ at 1 kHz were not included in the analysis.

### 4.7 Electromechanical Characterization

MEA stretching was achieved using a custom‐built stretcher (Figure S6) controlled by Arduino IDE software, with a speed of 100 µm/s and a step size of 306 µm (corresponding to 2.5% strain). At each elongation step, the electrodes were electrochemically characterized by recording EIS as previously described. In case of MEAs mounted on the multimodal platform, EIS was performed in a two‐electrode setup, using the reference electrodes of the MEA as the counter electrode, and filling the fluidic channel with PBS.

### 4.8 Spheroid Seeding on Kirigami‐Based MEAs and Maintenance

Brain spheroids were cultured following the protocol described by Govindan et al. [39]. At least 8 weeks after formation, the spheroids were transferred onto hollow, porous PTFE membranes placed in a 6‐well transwell insert to facilitate handling. These PTFE membranes were subsequently flipped onto the MEA, bringing the spheroids in contact with the electrodes. After four days of maintenance on the MEA, the PTFE membrane was gently removed using tweezers. Cells were kept at 37°C in a humidified CO_2_ incubator, and cell culture medium was replaced every other day. To minimize the impact of any transient effects associated with the transfer procedure on our analysis, spheroids were maintained on the MEA for 5 days following PTFE membrane removal before electrophysiological recordings coupled with mechanical probing were performed. This recovery period was intended to allow the tissue to re‐establish stable functional activity, should the transfer process have induced any temporary disturbance.

### 4.9 Scanning Electron Microscopy of Spheroids on Kirigami‐Based MEAs

Spheroids were maintained on the MEAs for 8 days prior to imaging. To prepare the tissues for scanning electron microscopy, spheroids were washed twice with PBS and fixed in 4% paraformaldehyde for 2 h at room temperature. Following fixation, tissues were washed with PBS and stored in PBS at 5°C until further processing. For dehydration, tissues were subjected to a stepwise ethanol series directly within the biochip (70% ethanol, 90% ethanol, 100% ethanol; 15 min each). Dehydration was followed by a two‐step drying protocol using hexamethyldisilazane (HMDS). First, tissues were incubated in 50% HMDS in ethanol for 20 min (repeated twice), then in 100% HMDS for 20 min (repeated twice). Excess HMDS was discarded, and residual solvent was allowed to evaporate overnight at room temperature. The tissues were coated with a 10 nm Au layer by sputtering (AC 450, Alliance Concept) directly on the biochip, and imaging was performed using a scanning electron microscope (SU5000, Hitachi).

### 4.10 Immunostaining

Spheroids were maintained on the MEAs for 11–14 days before imaging. To prepare the tissues for staining, spheroids were washed twice with PBS and fixed in 4% paraformaldehyde for 45 min at room temperature. After fixation, the tissues were washed with PBS and stored in PBS at 5°C until staining. For permeabilization, tissues were incubated in 0.1% Triton X‐100 for 15 min at room temperature, followed by PBS washes and a 1‐hour blocking step in 1% bovine serum albumin diluted in PBS. Primary antibodies were applied overnight at 4°C in PBS: anti‐MAP2 (131 500, Invitrogen, mouse; dilution 1:500) and anti‐GFAP (Z0334, Dako, rabbit; dilution 1:250). The next day, tissues were washed with PBS and incubated with secondary antibodies for 1 h at room temperature in the dark: Alexa Fluor 488 (A21202, Invitrogen, anti‐mouse; dilution 1:500) and Alexa Fluor 647 (A21244, Life Technologies, anti‐rabbit; dilution 1:500). DAPI (022M4004V, Sigma) was added as a nuclear stain at a 1:1000 dilution. After final washes in PBS, samples were imaged directly within the biochip platform using a confocal microscope (ZEISS Axio Observer). Z‐stacks of the entire spheroid volume were captured at various stretch levels. Image processing was conducted using Fiji software [51], extracting max intensity projections along the Z axis.

### 4.11 Electrophysiological Recordings and Analysis

Electrophysiological recordings were conducted for 10 min inside the cell culture incubator, using a wired connection between the custom‐built PCB in the biochip and the recording system. Signals were amplified using two 16‐channel Digital Electrophysiology Stimulator/Amplifier Chips (RHS2116, Intan Technologies) and acquired using the Spike‐on‐Chip platform [37]. Data from 32 electrodes (a complete device) were sampled at 30 kHz, with an amplifier passband from 5 Hz to 7.5 kHz. Post‐acquisition signal processing was performed in Python 3.11.4. For each recording, the noise level of each channel (σ) was defined as an estimate of the standard deviation of the background noise [52] and calculated as $\sigma =median(\frac{\left|S(t)\right|}{0.6745})\mu V$, where *S(t)* is the band‐passed filtered signal at time *t*. Spike detection was performed using the *signal.find_peaks* function from the *scipy* library [53], considering both positive and negative spikes. Spikes were detected based on amplitude thresholding, with the threshold set as Thr = 6σ and a minimum inter‐spike interval of 500 µs imposed to prevent duplicate detection. Channels were classified as functional if σ < 10µV, and active if the firing rate exceeded 2 spikes/min. To identify distinct spike waveforms detected by individual channels, waveform features were first reduced in dimensionality using Principal Component Analysis, after which spikes were clustered using Density‐Based Spatial Clustering.

### 4.12 Live Imaging While Stretching

Spheroids were maintained on the MEAs for 9–10 days prior to imaging, with the PTFE membrane removed on day 4. For cytoplasmic membrane staining, spheroids were labeled using BioTracker 400 Blue Cytoplasmic Membrane Dye (SCT109, Sigma‐Aldrich). The dye was prepared in pre‐warmed cell culture media following the manufacturer's recommended ratios. The staining solution was added to the fluidic channel, with one drop applied directly to each spheroid. Spheroids were incubated at 37°C in staining solution for 1 h, followed by a wash in pre‐warmed cell culture media. After staining, the biochip was mounted onto the stretching platform and transferred to a fluorescent microscope (ZEISS Axio Observer) for imaging. Z‐stacks of the entire spheroid volume were captured at various stretch levels. Image processing was conducted using Fiji software [51], extracting max intensity projections along the Z axis. Spheroid lengths along the x and y axes were manually measured and used to calculate the corresponding strains.

### 4.13 Pendulum‐Actuated Stretcher for Fast Actuation

The platform consists of a stationary bottom plate to which the culturing biochip is fixed, a linear stage to define the total MEA elongation, and a vertical stand to which a striker arm is secured. The platform was designed to allow for a customizable range of displacements, velocities, and forces by adjusting four key parameters: the distance *d* of the linear stage, the mass *m*, the length *l*, and the initial angle *θ* of the striker arm (Figure S16a,b). The system was designed with its max configuration (*l*_max_ = 17.5 cm, *θ* = 90°) to reach a stretching speed of 1.85 m/s in line with the injuries in contact sports. Figure S16c illustrates the actuation mechanism of the platform. The process begins by setting the desired distance *d* using the linear stage, which will determine the overall elongation of the MEA. Next, the angle *θ* and the length *l* of the striker arm are adjusted to define the elongation velocity. During operation, the striker arm impacts a movable element connected to the stretchable MEAs through the biochip's movable component. This action rapidly stretches the MEAs. Following the impact, the striker arm rebounds, and a dedicated stopping mechanism halts the striker arm, avoiding a second elongation of the MEA.

### 4.14 Statistical Analysis

Statistical comparisons of electrophysiological activity before (baseline) and after stretch injury were performed using recordings from three spheroids, each sampled by eight electrodes (24 electrodes in total; Figure 4h,j and Table S2). The three spheroids were located on the same device and subjected to stretch simultaneously. Electrophysiological activity at each post‐injury time point (10 min, 1 h, and 2 h post‐injury) was compared independently with baseline activity. To maintain consistency with the analysis performed throughout the study, the primary statistical analysis was restricted to electrodes that were active (firing rate > 2 spikes/min) both at baseline and post‐injury (Table S3). Electrodes that were active at baseline but exhibited no detectable firing post‐injury were therefore excluded from the paired analysis. For each condition, electrodes that were active both at baseline and post‐injury were identified and paired. In the sham condition, 14 electrodes were active at both baseline and 10 min post‐injury, 18 at both baseline and 1 h post‐injury, and 17 at both baseline and 2 h post‐injury. In the test condition, 10 were active at both baseline and 10 min post‐injury, 18 at both baseline and 1 h post‐injury, and 21 at both baseline and 2 h post‐injury. For each comparison, baseline and post‐injury firing rates were compared using a paired two‐tailed *t*‐test (Python 3.11.4). Because the cessation of firing following stretching may itself represent an injury‐related response, a complementary analysis was performed including all functional electrodes (σ < 10µV), including those whose firing rate decreased to zero after injury. This secondary analysis followed the same paired‐comparison approach as the primary analysis and is reported in Table S4.

## Author Contributions

**Eleonora Martinelli**: investigation, conceptualization, Writing – original draft, methodology, validation, visualization, Writing – review and editing, formal analysis, supervision. **Ivan Furfaro**: conceptualization, validation, methodology. **Flavius Timis**: validation, data curation, formal analysis. **João Marques**: formal analysis, data curation, investigation. **Tala El Kaissi**: formal analysis, data curation, investigation. **Desirée Maulà**: investigation, conceptualization, validation, methodology, supervision. **Laurine Kolly**: conceptualization, formal analysis, validation. **Julian Bär**: methodology, formal analysis, data curation, software. **Sina Röllin**: software, validation, visualization. **Scott Erickson**: investigation, supervision, formal analysis, methodology. **Laetitia Nikles**: validation, data curation. **Benoît Delaup**: methodology, data curation. **Camille Delgrange**: data curation. **Loris Gomez Baisac**: methodology, formal analysis, data curation. **Luc Stoppini**: conceptualization, methodology, supervision. **Adrien Roux**: methodology, supervision. **Giuseppe Schiavone**: conceptualization, investigation, methodology, writing – original draft, writing – review and editing, supervision. **Stéphanie P. Lacour**: conceptualization, funding acquisition, methodology, visualization, writing – review and editing, project administration, supervision, resources.

## Funding

This work was supported by SNSF Grant 200021_219663.

## Conflicts of Interest

The authors declare no conflicts of interest.

## Supporting information




**Supporting File**: adhm71788‐sup‐0001‐SuppMat.docx.

## Data Availability

Data are available at https://doi.org/10.5281/zenodo.19095706.
