# Supplementary material for: Stretchable Microelectrode Arrays for Electro‐Mechanical Probing of Brain Spheroids
Source: Adv Healthc Mater. 2026 Oct 6;15(40):e71788. doi: 10.1002/adhm.71788 (PMC13712204; doi:10.1002/adhm.71788)
Supplement: Supplementary file 1 [file ADHM-15-0-s001.docx]

Supporting Information

**Stretchable microelectrode arrays to electro-mechanically probe brain spheroids**

*Eleonora Martinelli, Ivan Furfaro, Flavius Timis, João Marques, Tala El Kaissi, Desirée Maulà, Laurine Kolly, Julian Bär, Sina Röllin, Scott Erickson, Laetitia Nikles, Benoît Delaup, Camille Delgrange, Loris Gomez Baisac, Luc Stoppini, Adrien Roux, Giuseppe Schiavone, and Stéphanie P. Lacour**

* E-mail: stephanie.lacour@epfl.ch

**Table S1:** Main works measuring neural activity with MEAs *in vitro* for TBI research.

| **Tissue** | **Injury system** | **MEA type** | **Injury parameters** | **Electrophysiology signal** | **Time points** | **Ref** |
| --- | --- | --- | --- | --- | --- | --- |
| Neural organoids (human) | Injector | Flexible  MEA | Jet distance, duration | Spontaneous | 0h, 1h, 2h, 24h, 48h | ^1^ |
| Neural organoids (human) | Pressure chamber | Rigid MEA | Pressure amplitude, frequency | Spontaneous | 1h, 24h | ^2^ |
| Cellular monolayer (mouse) | Ballistic pendulum | Rigid MEA | Tangential acceleration, impact number | Spontaneous | Up to 24h | ^3^ |
| Organotypic slice (rat) | Injector | Rigid MEA | Pressure, duration, impulse | Spontaneous, Evoked | 4-6d | ^4^ |
| Organotypic slice (rat) | Equibiaxial stretcher | Rigid MEA | Strain amplitude, rate | Spontaneous, evoked | 4-6d | ^5^ |
| Organotypic slice (rat) | Equibiaxial stretcher | Intrinsically stretchable MEA | Strain amplitude, rate | Spontaneous | 0h-24h | ^6^ |
| Organotypic slice (rat) | Equibiaxial stretcher | Rigid MEA | Strain amplitude, rate | Spontaneous, Evoked | 4-6d | ^7^ |
| Organotypic slice (rat) | Equibiaxial stretcher | Rigid MEA | Strain amplitude, rate | Evoked | 2d, 4d, 6d | ^8^ |
| Organotypic slice (rat) | Equibiaxial stretcher | Intrinsically stretchable MEA | Strain amplitude, rate | Evoked | 0h | ^9^ |
| Organotypic slice (rat) | Equibiaxial stretcher | Intrinsically stretchable MEA | Strain amplitude, rate | Evoked | 0h, 4-9d | ^10^ |
| Organotypic slice (rat) | Equibiaxial stretcher | Intrinsically stretchable MEA | Strain amplitude, rate | Evoked | 0h, 4-9d | ^11^ |
| Organotypic slice (rat) | Equibiaxial stretcher | Rigid MEA | Strain amplitude, rate | Evoked | 6d | ^12^ |
| Organotypic slice (rat) | Equibiaxial stretcher | Intrinsically stretchable MEA | - | - | - | ^13^ |
| Cellular monolayer (rat) | Shearing device | Rigid MEA | Shear stress magnitude, duration, rise time | Spontaneous | 10min | ^14^ |

**Figure S1: Fabrication process flow of kirigami-based MEAs.** Steps 1–11 illustrate the microfabrication steps for creating the MEA, followed by steps 12–13, which detail the integration of the MEAs onto custom-made PCBs.

**Figure S2: Mechanical behaviour of PI films.** **a** Schematic of plain PI samples used for mechanical testing with a thickness of 2 μm. **b** Stress-strain response of plain PI under uniaxial loading to failure for n = 3 samples. **c** Dogbone dimensions. The kirigami-patterned area is limited to the dogbone part of the sample (orange). **d** Schematic of kirigami-patterned PI samples used for mechanical testing, with a thickness of 2 μm. **e** Stress-strain response of kirigami-patterned PI under uniaxial loading to failure. n = 1 sample. **f** Cyclic stress-strain response of kirigami-patterned PI under progressively increasing uniaxial strain levels (10%, 15%, and 20%). n = 1 sample per strain level. **g** In situ SEM imaging of kirigami-patterned PI under increasing strain, highlighting the onset of out-of-plane deformation. Tilt ~30°.

**Figure S3: SEM while stretching.** **a** Schematics of the system. Created with BioRender.com **b** Pictures of the custom-made holder for reliable assembly on the stretcher. First, each side of the MEA is secured to two clamps with RTV (step 1). A bridge support is then fixed to the clamps (step 2). The MEA and clamps are removed from the holder (step 3) and mounted onto the stretcher (step 4). Finally, the support is removed (step 5), the stiffener frame is cut, and stretching can begin (step 6).

**Figure S4:** **Strain distribution and electrode positioning on the PI dogbone.** **a** FEM simulation of the strain distribution along the PI dogbone under 5% overall strain. The dashed white line represents the middle arc of the dogbone along the y-axis. **b** Simulated strain extracted along the y-axis middle arc under 5% overall strain. **c** Schematics of the dogbone sample geometry, with the electrodes positioned along the midline. The kirigami-patterned area is limited to the dogbone section (orange)

**Figure S5: RJD analysis pipeline.** Reference SEM images are acquired at 0% applied strain and corresponding target images at increasing strain levels, then converted to binary masks to segment the kirigami mesh. Mesh joints are detected as strand intersections (red dots) and tracked across strain conditions (color-coded for verification). A grid-based neighborhood assignment is used to identify the up/down neighbors of each joint. For each joint, inter-joint distances are computed in both reference and target images, and the normalized change in these distances is used to calculate RJD, providing a spatial quantification of local mesh deformation.

**Figure S6: Custom electromechanical setup for impedance spectroscopy under mechanical strain.** Schematic of the custom-built system used to characterize the kirigami-based MEAs during stretching. The PCB-mounted device is integrated into a motorized tensile stage (displacement rate: 100 µm/s), controlled via an external driver and computer interface. During testing, the kirigami-based MEAs are immersed in PBS together with a Pt counter electrode and an Ag/AgCl reference electrode. Electrical measurements are performed through a potentiostat connected to the PCB, enabling simultaneous application of mechanical strain and recording of electrochemical impedance spectroscopy. Created with BioRender.com.

**Figure S7: Optimization of the electrode geometry. a** Schematic of the six electrode geometries (condition C1 to condition C6) used in the optimization study, varying electrode shape (circular or rectangular), width of the Pt (w_Pt_) and PI (w_PI_) layers (w_PI_ = w_Pt_ =2 μm, w_PI_ = 3w_Pt_, w_PI_ = w_Pt_ = 6 μm), and Pt layer thickness (t_Pt_ = 100 nm, 200 nm, or 300 nm). **b** Maximum strain modeled in the Pt layer at 10% ɛ_app_ across the different geometries. **c** SEM images of the unoptimized (C1) and optimized electrode (C5) after fabrication on the Al-coated wafer. False colors represent the different materials. **d** RJD as a function of ɛ_app_. e) |ΔZ| at 1 kHz as a function of ɛ_app_ for the unoptimized (C1) and optimized (C5) geometries. Data of C5 from Figure 1i. **e** Variation in |ΔZ| at 1 kHz as a function of ɛ_app_ for the unoptimized (C1) and optimized (C5) geometries, for 24 stretched electrodes (3 MEAs of 8 electrodes each, orange), compared to unstretched controls (1 MEA, blue). Data of C5 from Figure 1k. Error bars indicate 95% CI of the mean.

**Figure S8: Dependence of RJD on electrode spacing.** **a** Schematics and corresponding SEM images of kirigami-patterned electrodes arranged in grid layouts with progressively reduced electrode spacing (left to right) at 0% ɛ_app_ (top row) and 15% ɛ_app_ (bottom row). **b** RJD maps as a function of electrode spacing at 15% ɛ_app_ (left to right). For each RJD map, the RJD profiles along line A (y-axis) and line B (x-axis) are shown as a function of the applied strain.

**Figure S9: Dependence of RJD on electrode diameter.** **a** Schematics (top) and corresponding SEM images (bottom) of samples consisting of four kirigami-patterned electrodes with progressively increased electrode diameter (left to right). **b** RJD maps at 10% ɛ_app_ as a function of the electrode diameter (left to right). **c** RJD profiles along the x-axis extracted from each RJD map. **d** RJD profiles along the y-axis extracted from each RJD map.

**Figure S10: Joint detection and tracking across the kirigami-based MEA surface.** Top row: SEM images of a kirigami-based MEA acquired at increasing strain levels. False colors indicate recording electrodes (blue, n=8) and ground electrodes (green, n=2). Scale bar 70 μm. Second row: corresponding binary images. Third row: detected joints represented as red dots. Bottom row: joint tracking across strain conditions, with corresponding joints color-coded to visualize point-to-point matching.

**Figure S11: Brain spheroid seeding on kirigami-based MEAs.** **a** Schematic of the procedure for precise spheroid seeding on stretchable MEAs: spheroids are first pipetted onto a hollow PTFE membrane (1), which is then carefully lifted with tweezers and flipped upside down (2-3), to transfer the spheroid onto the MEA (3-4). After four days of culture, the PTFE membrane is removed using tweezers, leaving the tissue adhered to the MEA (5-6). Created with BioRender.com. **b** Photographs illustrating the step-by-step seeding process. Scale bar: 4mm. **c** Brightfield images showing a spheroid on a stretchable MEA before and after removal of the PTFE membrane.

**Figure S12: Spheroid staining on kirigami-based MEAs.** **a-b** Immunostaining showing GFAP+ glial cell outgrowth (red, arrowheads) around the spheroid contour at day 11 post-seeding on the kirigami-based MEAs.

**Figure S13: Manual stretcher for slow, linear mechanical actuation.** **a** Photograph of the biochip fixed to the precision linear stage and inserted in an alignment support, enabling precise component alignment during stretching. **b** Step-by-step process for mounting the biochip on the stretcher and positioning it on the microscope: the biochip is inserted into the alignment support, aligned, and fixed to the linear stretcher. It is then removed from the alignment support and placed onto the microscope stage. Stretching can be performed either within the alignment support or directly on the microscope stage.

**Figure S14: Spheroid deformation upon stretch.** Live cell staining of spheroids on kirigami-based MEAs upon stretching using BioTracker^TM^ cell membrane dye (n_biochip_=1, n_spheroid_=3).

**Figure S15: Spontaneous electrophysiological activity during mechanical actuation.** **a** Timeline of the experimental protocol from seeding (day 0) to mechanical actuation (day 9). **b** Strain profile as a function of time during mechanical actuation on day 9. Electrophysiological recordings were performed at each strain step and lasted 10 min, for a total experiment duration of approximately 1h. **c** Raster plots of the spontaneous electrophysiological activity of two spheroids as a function of the applied strain, at day 9 post-seeding (n = 2 MEAs, 2 spheroids). **d** Number of functional electrodes (noise level < 10 μV) per MEA as a function of the applied strain. **e** Noise level of the functional electrodes as a function of the strain level. The shaded area indicates 95% CI around the mean. **f** Number of active electrodes (spike rate > 2 spikes/min) per MEA as a function of the applied strain.

**Figure S16: Pendulum-actuated stretcher for fast, linear mechanical actuation.** **a** Schematic of the pendulum-actuated stretcher platform: the movable element of the biochip fluidic channel is fixed to a linear stage, operated by the striker arm of a pendulum. Multiple parameters can be varied to control the speed and elongation of the MEAs: the elongation distance *d*, the striker arm angle *θ*, the arm length *l*, and mass *m*. **b** Photograph of the overall platform. **c** Step-by-step actuation process. During actuation, the striker arm impacts a movable component linked to the MEA (1.), rapidly stretching it up to a set distance *d* (2.). After impact, the striker arm rebounds, and a stopping mechanism prevents a second elongation (3.).

**Figure S17: Spontaneous electrophysiological activity following fast mechanical actuation. a** Timeline of the experimental protocol from seeding (day 0) to mechanical actuation (day 10). At day 10 post-seeding, fast mechanical actuation was delivered using a custom-made pendulum system (10 impacts at 7.5% ɛ_app_ spaced ~30s apart). Spontaneous activity was recorded for 10 min at baseline (pre-stimulation) and after deformation at 10 min, 1 h, and 2 h. A sham condition was performed at day 9, during which the biochip was removed from and returned to the incubator without mechanical stimulation to assess the effect of handling and temperature changes. **b** Raster plots of the spontaneous electrophysiological activity recorded during the sham and test conditions (n = 3 MEAs, 3 spheroids). **c** Number of functional electrodes (noise level < 10 μV) per MEA during sham and test phases (n = 3 MEAs, 3 spheroids). Error bars indicate 95% CI of the mean. **d** Number of active electrodes (spike rate > 2 spikes/min) per spheroid during sham and test phases (n = 3 MEAs, 3 spheroids).

**Table S2:** Spontaneous electrophysiological activity following fast mechanical actuation: structure of the analysed electrophysiological dataset.

| **Electrode**  **(1 to 24)** | **Spheroid**  **(A, B, C)** | **Sham/Test** | **Time**  **Post-injury** | **Firing Rate (Hz)** | **Functional**  **(σ < 10μV)** | **Active**  **(firing rate > 2 spikes/min)** |
| --- | --- | --- | --- | --- | --- | --- |
| 1 | A | Sham | Baseline | 5.4 | True | True |
| 1 | A | Sham | 10min | 1.6 | True | True |
| 1 | A | Sham | 1h | 5.3 | True | True |
| 1 | A | Sham | 2h | 16.7 | True | True |
| 1 | A | Test | Baseline | 10.2 | True | True |
| 1 | A | Test | 10min | 0.0 | True | False |
| 1 | A | Test | 1h | 2.5 | True | True |
| 1 | A | Test | 2h | 16.7 | True | True |
| … | … | … | … | … | … | … |
| 24 | C | Sham | Baseline | 1.8 | True | True |
| 24 | C | Sham | 10min | 1.2 | True | True |
| 24 | C | Sham | 1h | 5.2 | True | True |
| 24 | C | Sham | 2h | 6.8 | True | True |
| 24 | C | Test | Baseline | 5.5 | True | True |
| 24 | C | Test | 10min | 0.6 | True | True |
| 24 | C | Test | 1h | 4.0 | True | True |
| 24 | C | Test | 2h | 7.5 | True | True |

**Table S3:** Statistical analysis including active electrodes, with 18 active electrodes at baseline in the sham condition and 21 in the test condition. *p<0.05.

| **Sham/Test** | **Time**  **Post-Injury** | **Tot Active Electrodes** | **Paired Electrodes** | **Mean**  **Baseline (Hz)** | **Mean**  **Post-injury (Hz)** | **Paired**  **two-tailed t-test p** |
| --- | --- | --- | --- | --- | --- | --- |
| Sham | 10 min | 15 | 14 | 3.3 | 2.3 | 0.2321 |
| Sham | 1h | 20 | 18 | 3.3 | 4.6 | 0.0805 |
| Sham | 2h | 19 | 17 | 3.5 | 7.4 | 0.0489 [*] |
| Test | 10 min | 10 | 10 | 4.8 | 0.7 | 0.0349 [*] |
| Test | 1h | 18 | 18 | 6.7 | 3.3 | 0.0913 |
| Test | 2h | 21 | 21 | 6.6 | 6.3 | 0.8679 |

**Table S4:** Statistical analysis including functional electrodes, with 19 functional electrodes at baseline in the sham condition and 22 in the test condition. *p<0.05, **p<0.01.

| **Sham/Test** | **Time**  **Post-injury** | **Tot Functional Electrodes** | **Paired Electrodes** | **Mean**  **Baseline (Hz)** | **Mean**  **Post-injury (Hz)** | **Paired two-tailed t-test p** |
| --- | --- | --- | --- | --- | --- | --- |
| Sham | 10 min | 21 | 19 | 3.1 | 1.7 | 0.0655 |
| Sham | 1h | 21 | 19 | 3.1 | 4.4 | 0.08059 |
| Sham | 2h | 21 | 19 | 3.1 | 6.6 | 0.04985 [*] |
| Test | 10 min | 20 | 20 | 6.7 | 0.3 | 0.003405 [**] |
| Test | 1h | 21 | 21 | 6.6 | 2.8 | 0.03883 [*] |
| Test | 2h | 21 | 21 | 6.6 | 6.3 | 0.8679 |

**References**

1.	Loussert-Fonta, C., Stoppini, L., Neuenschwander, Y., Righini, O., Prim, D., Schmidt, C., Heuschkel, M. O., Gomez Baisac, L., Jovic´, M., Pfeifer, M. E., Extermann, J. & Roux, A. Opening the black box of traumatic brain injury: a holistic approach combining human 3D neural tissue and an in vitro traumatic brain injury induction device. *Front. Neurosci.* **17**, (2023).

2.	Silvosa, M. J., Mercado, N. R., Merlock, N., Vidhate, S., Mejia-Alvarez, R., Yuan, T. T., Willis, A. M. & Lybrand, Z. R. Understanding Primary Blast Injury: High Frequency Pressure Acutely Disrupts Neuronal Network Dynamics in Cerebral Organoids. *J. Neurotrauma* **39**, 1575–1590 (2022).

3.	Rogers, E. A. & Gross, G. W. Simultaneous electrophysiological and morphological assessment of functional damage to neural networks in vitro after 30–300 g impacts. *Sci. Rep.* **9**, 14994 (2019).

4.	Vogel, E. W., Effgen, G. B., Patel, T. P., Meaney, D. F., Bass, C. R. “Dale” & Morrison, B. Isolated Primary Blast Inhibits Long-Term Potentiation in Organotypic Hippocampal Slice Cultures. *J. Neurotrauma* **33**, 652–661 (2016).

5.	Kang, W. H. & Morrison, B. Predicting changes in cortical electrophysiological function after in vitro traumatic brain injury. *Biomech. Model. Mechanobiol.* **14**, 1033–1044 (2015).

6.	Kang, W. H., Cao, W., Graudejus, O., Patel, T. P., Wagner, S., Meaney, D. F. & Morrison, B. Alterations in Hippocampal Network Activity after In Vitro Traumatic Brain Injury. *J. Neurotrauma* **32**, 1011–1019 (2015).

7.	Kang, W. H. & Morrison, B. Functional tolerance to mechanical deformation developed from organotypic hippocampal slice cultures. *Biomech. Model. Mechanobiol.* **14**, 561–575 (2015).

8.	Yu, Z. & Morrison, B. Experimental Mild Traumatic Brain Injury Induces Functional Alteration of the Developing Hippocampus. *J. Neurophysiol.* **103**, 499–510 (2010).

9.	Lacour, S. P., Benmerah, S., Tarte, E., FitzGerald, J., Serra, J., McMahon, S., Fawcett, J., Graudejus, O., Yu, Z. & Morrison, B. Flexible and stretchable micro-electrodes for in vitro and in vivo neural interfaces. *Med. Biol. Eng. Comput.* **48**, 945–954 (2010).

10.	Yu, Z., Graudejus, O., Tsay, C., Lacour, S. P., Wagner, S. & Morrison, B. Monitoring Hippocampus Electrical Activity *In Vitro* on an Elastically Deformable Microelectrode Array. *J. Neurotrauma* **26**, 1135–1145 (2009).

11.	Yu, Z., Graudejus, O., Lacour, S. P., Wagner, S. & Morrison, B. Neural sensing of electrical activity with stretchable microelectrode arrays. *Annu. Int. Conf. IEEE Eng. Med. Biol. Soc. IEEE Eng. Med. Biol. Soc. Annu. Int. Conf.* **2009**, 4210–4213 (2009).

12.	Yu, Z., Elkin, B. S. & Morrison, B. Quantification of functional aalterations after in vitro traumatic brain injury. in *2009 Annual International Conference of the IEEE Engineering in Medicine and Biology Society* 1135–1138 (2009). doi:10.1109/IEMBS.2009.5332381.

13.	Yu, Z., Tsay, C., Lacour, S. P., Wagner, S. & Morrison, B. Stretchable microelectrode arrays a tool for discovering mechanisms of functional deficits underlying traumatic brain injury and interfacing neurons with neuroprosthetics. in *2006 International Conference of the IEEE Engineering in Medicine and Biology Society* 6732–6735 (IEEE, New York, NY, 2006). doi:10.1109/IEMBS.2006.260933.

14.	Prado, G. R., Ross, J. D., DeWeerth, S. P. & LaPlaca, M. C. Mechanical trauma induces immediate changes in neuronal network activity. *J. Neural Eng.* **2**, 148–158 (2005).
